# Adults with celiac disease exhibit overexpression of endogenous retroviruses, TRIM28, and SETDB1 despite gluten-free diet

**DOI:** 10.1016/j.virusres.2025.199613

**Published:** 2025-08-05

**Authors:** Pier-Angelo Tovo, Angelo Armandi, Mauro Bruno, Gian Paolo Caviglia, Paola Montanari, Demis Pitoni, Cristina Calvi, Simone Frara, Eleonora Dileo, Stefano Gambarino, Ilaria Galliano, Davide Giuseppe Ribaldone, Massimiliano Bergallo

**Affiliations:** a Department of Public Health and Pediatric Sciences, University of Turin, Piazza Polonia 94 10126 Turin, Italy; b Department of Medical Sciences, Division of Gastroenterology and Hepatology, University of Turin, Corso Achille Mario Dogliotti 14 10123, Turin, Italy; c Pediatric Laboratory, Department of Public Health and Pediatric Sciences, University of Turin, Regina Margherita Children's Hospital, Piazza Polonia 94 10126 Turin, Italy

**Keywords:** Celiac disease, Human endogenous retroviruses, TRIM28, SETDB1, GFD, epigenetics

## Abstract

Celiac disease (CeD) is a disorder due to abnormal immune response to gluten protein in individuals with predisposing genotypes. Its origin is not fully understood. Human endogenous retroviruses (HERVs) derive from ancestral infections of germinal cells and represent 8 % of the human DNA. They are mostly inactive, but some can be activated. Their aberrant expressions are associated with inflammatory and immune-mediated diseases. HERV transcription is modulated by TRIM28 and SETDB1, which are also directly implicated in epigenetic processes and modulation of the immune response. We reported HERV overexpressions in CeD children at diagnosis. In the current prospective study, using a PCR real-time Taqman amplification assay, we explored the transcription levels of HERV-H-pol, -HERV-K-pol, and HERV-W-pol, of syncytin 1 (SYN1), SYN2, and HERV-W-env, as well as of TRIM28 and SETDB1 in the whole blood from 51 adults with CeD after years of good compliance with gluten-free diet (GFD) as compared to healthy controls (HC) of similar age.

The transcriptional levels of every HERV and of TRIM28/SETDB1 were significantly higher in CeD patients than in HC (constantly with *p* < 0.0001). Positive correlations were found between the RNA levels of TRIM28 or SETDB1 and HERVs in CeD patients. The upregulation of HERVs, TRIM28, and SETDB1 and their positive correlations are suggestive clues of their contribution to the pathophysiology of CeD and might justify the persistent risk of developing, despite GFD, autoimmune diseases, neuropsychiatric disturbances, and cancers, all disorders characterized by enhanced HERV expressions and epigenetic alterations.

## Highlights

• Celiac disease is immune-mediated and triggered by gluten in predisposed people.
• HERVs are viral DNA remnants linked to immune and inflammatory disorders.
• The study analyzed HERVs, TRIM28, SETDB1 in 51 celiac adults on gluten-free diet.
• CeD patients showed higher HERV and gene expression vs. healthy controls.
• Persistent HERV activation may raise long-term autoimmune and cancer risks.

## 1 Introduction

Celiac disease (CeD) is a permanent immune-mediated enteropathy, with heterogeneous clinical manifestations occurring in about 1 % of the global population (Fasano and Catasi, 2012). HLA-DQ2 and HLA-DQ8 are predisposing genotypes, while the gluten protein present in wheat, barley, and rye represents the triggering element. About one-third-of the general population is however HLA-DQ2/DQ8 carrier, but only a fraction develops the disease. Therefore, other pathogenetic factors are involved. Strict adherence to gluten-free diet (GFD) is the only effective treatment for CeD, although up to 25–50 % of adults still manifest symptoms or develop comorbidities typically associated with CeD. Hence, additional and alternative treatments, profiling CeD-related transcriptional changes, and the role of the noncoding genome are new fields that are currently being investigated.

Human endogenous retroviruses (HERVs) represent approximately 8 % of human DNA. They derive from ancestral infections of germinal cells and subsequent transmission to future generations through Mendelian inheritance (Johnson, 2019). HERVs retain their retroviral structure with three principal genes: group associated antigens (gag), polymerase (pol), and envelope (env), flanked between two regulatory long terminal repeats (LTRs) (Johnson, 2019). The accumulated mutations over the millennia made most HERVs inactive. Some maintain however the ability to be transcribed and a few encode proteins that are co-opted for pivotal physiological functions during the intrauterine life, such as the Syncytin (SYN) 1 and 2 (SYN 2), which contribute to the placental syncytiotrophoblast formation and to materno-fetal immunotolerance (Garcia‐Montojo et al., 2020; Lokossou et al., 2020). HERVs can act as promoters of neighboring host genes and influence innate and adaptive immune responses (Chuong et al., 2016). There are mutual interactions between HERVs and inflammatory cytokines, and retroviral antigens can trigger specific reactivity and molecular mimicry with tissue antigens (Balada et al., 2010; Grandi and Tramontano, 2018; Rolland et al., 2006). A body of clinical and experimental studies has shown a clear association between heightened HERV expressions and immune‐mediated diseases (Balada et al., 2010; Curtin et al., 2018; Grandi and Tramontano, 2018; Rolland et al., 2006), including gastrointestinal disorders, such as inflammatory bowel disease (Tovo et al., 2024), irritable bowel syndrome (Tovo et al., 2025), and food allergy (Tovo et al., 2022). HERVs also have a reciprocal influence on microbiota and their overexpressions promote microbiota-driven intestinal inflammation (Lima-Junior et al., 2021).

HERV transcription is tightly regulated by epigenetic mechanisms via DNA methylation and histone tails variations. Tripartite motif-containing 28 (TRIM28) and SET domain bifurcated histone lysine methyltransferase 1 (SETDB1) are cellular genes playing essential roles in modulating HERV expression (Adoue et al., 2019; Turelli et al., 2014). Both are also directly implicated in the regulation of the immune response and epigenetic processes (Adoue et al., 2019; Manghera and Douville, 2013), which contribute to the development of CeD (Ghosh et al., 2022; Gnodi et al., 2022).

Steatotic liver disease (SLD) is a common comorbidity in CeD patients on GFD (Cazac et al., 2024). HERVs and SETDB1 have been implicated in the pathogenesis of liver damage (Wang et al., 2022; Weber et al., 2021), but no data are available on their expressions in CeD patients with SLD.

We demonstrated that the RNA levels of the pol genes of HERV-H, -K, and -W, the three most studied retroviral families, were significantly upregulated in peripheral blood of children with CeD at the time of diagnosis, when they were daily exposed to gluten-containing foods (Tovo et al., 2021). Whether this retroviral upregulation 1) involves other HERVs, 2) is present also in adults, 3) is linked to TRIM28 and SETDB1 alterations, 4) persists or disappears in patients on GFD, and 5) is more marked in CeD patients with SLD, remain unanswered questions. Therefore, the aims of this study were to assess and compare the transcriptional levels of HERV-H-pol, HERV-K-pol, and HERV-W-pol, of SYN1, SYN2, whose proteins strongly alter the immune response (Garcia‐Montojo et al., 2020; Lokossou et al., 2020), and HERV-W-env, that can trigger potent inflammatory reactions (Rollland et al., 2006), as well as of TRIM28 and SETDB1 in the whole blood of adults affected by CeD on GFD, with and without liver steatosis, and in healthy controls (HC) of similar age and gender distribution.

## 2 Materials and methods

### 2.1 Study populations

Patients with CeD regularly follow-up at the Division of Gastroenterology and Hepatology, University of Turin, Turin, Italy, on GFD for at least 12 months were prospectively enrolled in the study. The diagnosis of CeD was based on positive tissue transglutaminase (tTG)-IgA and Marsh 1–3 on duodenal biopsy (Rubio-Tapia et al., 2023). Every patient underwent full clinical and biochemical evaluation at time of blood sample collection. The adherence to the GFD was verified using the Biagi’s questionnaire (Biagi et al., 2009). Exclusion criteria included refractory CeD, persistence of intestinal symptoms and/or of anti-tTG-IgA, ongoing or suspected pregnancy, breastfeeding, daily alcohol consumption exceeding 20 gr/daily for women and 30 gr/daily for men, use of potential steatogenic medications, such as anastrozole, corticosteroids, and cytotoxic agents, and any chronic liver disease. All patients underwent non-invasive assessment of steatosis and fibrosis by vibration controlled transient elastography (Fibroscan F5330, Echosens, France). Liver steatosis was defined by Controlled Attenuation Parameter (CAP) > 247 dB/m (steatosis grade 1: 248–277 dB/m; steatosis grade 2: 278–299 dB/m; steatosis grade 3: >299 dB/m). Liver fibrosis was defined by Liver Stiffness Measurement (LSM) > 7 kPa. Hepatic damage was defined by the presence of any grade of liver steatosis or fibrosis.

Healthy volunteers (blood donors) were the control group. These were chosen with age and gender comparable to those of the patients, since the females-to-males ratio among CeD subjects is usually 2:1 (Lebwohl et al., 2018).

### 2.2 Blood sample storage

Peripheral blood samples from both patients and healthy controls were mixed with RNAPro Solution (Biomole, Turin, Italy) to preserve RNA integrity and subsequently stored at −80 °C until RNA extraction. Collection, preservation, storage, and processing procedures were identical for both patients and control group to ensure consistency and minimize pre-analytical variability.

### 2.3 Total RNA extraction

The method used to measure the transcription levels of each target gene has previously been described in detail (Tovo et al., 2021, 2022, 2024; Tovo et al., 2025). Briefly, total RNA was extracted through the Maxwell automated extractor, using the RNA Blood Kit (Promega, Madison, WI, USA), which includes DNase treatment. RNA extracts were also directly amplified to rule out any DNA contamination. RNA concentration and purity were assessed by UV spectroscopy, measuring absorbance at 260 and 280 nm (SimpliNano spectrophotometer, Biochrom US, Holliston, MA, USA). The RNAs were stored at −80 °C until use.

### 2.4 Reverse transcription

Four hundred nanograms of total RNA were reverse-transcribed in a 20 µL reaction mixture containing 2 µL of a 10 × buffer, 4.8 µL of 25 mM MgCl2, 2 µL ImProm-II reverse transcriptase (Promega), 1 µL of RNase inhibitor (20 U/L), 0.4 µL of 250 µM random hexamers (Promega), 2 µL of 100 mM dNTP mix (Promega), and nuclease-free water. The reverse transcription was carried out in a GeneAmp PCR system 9700 Thermal Cycle (Applied Biosystems, Foster City, CA, USA): 5 min at 25 °C, 60 min at 42 °C, followed by 15 min at 70 °C for enzyme inactivation. The cDNAs were stored at −20 °C until the final analysis.

### 2.5 Transcription levels of HERVs and TRIM8/SETDB1 by a real-time PCR assay

The relative expression (RE) levels of pol genes from HERV-H, HERV-K, and HERV-W; of env genes from SYN1, SYN2, and HERV-W; as well as of TRIM28/SETDB1 were measured using the primers and probes previously reported (Tovo et al. 2021; Tovo et al., 2022, 2024, 2025). GAPDH was selected as reference gene. Briefly, 40 ng of cDNA was amplified in a 20 µL reaction containing 2.5 U goTaQ MaterMix (Promega), 1.25 mmol/L MgCl2, 500 nmol of specific primers, and 200 nmol of specific probes. All the amplifications were carried out in a 96-well plate: initial denaturation at 95 °C for 10 min, 45 cycles at 95 °C for 15 s, and at 60 °C for 1 min. Each sample was analyzed in triplicate. The relative expression (RE) of target gene transcripts was carried out according to the 2−∆∆Ct method (Livak and Douville, 2001). Briefly, the Ct value of each target gene was first normalized to the Ct value of the housekeeping gene (GAPDH) to obtain the ∆Ct. Then, this ∆Ct value was calibrated by subtracting the median ∆Ct of the same gene obtained from a pool of HC. Since we measured Ct for every target in all samples, we argue that our methods were suitable for HERV and TRIM28/SETDB1 quantifications.

### 2.6 Statistical analysis

The Mann–Whitney test was used to evaluate differences in the expression of each target gene between CeD patients and HC. Spearman correlation test was used to evaluate the correlations between the transcript levels of TRIM28 or SETDB1 and those of single HERV sequences. Statistical analyses were performed using the Prism 7 software (GraphPad Software); *p* < 0.05 was taken to be statistically significant.

## 3 Results

### 3.1 Study populations

Fifty-one patients affected by CeD and 85 HC were enrolled in the study. The characteristics of the patients and control subjects are detailed in Table 1. The median age of CeD patients was comparable to that of HC (*p* = 0.1866). As expected, CeD was more prevalent in females (Biagi et al., 2009). A similar percentage of females was present in HC (Table 1).

**Table 1:** Demographics and clinical characteristics of patients with celiac disease (CeD) and healthy controls (HC).

	CeD *n* = 51	HC *n* = 85
*Median age* (years)	49.8	43.6
(IQR)	(31.4 – 54.4)	(35.8 – 61.8)
*Females* n ( %)	41 (80.0)	66 (77.6)
*Comorbidities*		
Hepatic steatosis*	13	
Grade 1	10	
Grade 2	–	
Grade 3	3	
Hepatic fibrosis*	2	
*Type 2 diabetes*	2	
*Duration of gluten-free diet*		
median (months)	177	
(IQR)	(105 – 250)	

n: number; IQR: interquartile range, expressed as 25 and 75 quartile values.

⁎ diagnosed through fibroscan.

All CeD patients maintained strict GFD according to the Biagi’s questionnaire, were anti-tTG-IgA negative, and declared no intestinal symptoms.

The expression levels of the housekeeping gene (GAPDH) were comparable between CeD patients and healthy controls (*p* = 0.9329).

The median values of every target gene were similar between males and females both among HC (Supplementary Figure S1) and CeD patients (Supplementary Figure S2).

### 3.2 Transcription levels of HERV-H-pol, HERV-K-pol, and HERV-W-pol in the whole blood of 51 patients with celiac disease (CeD) and healthy controls (HC)

**Fig. 1:**
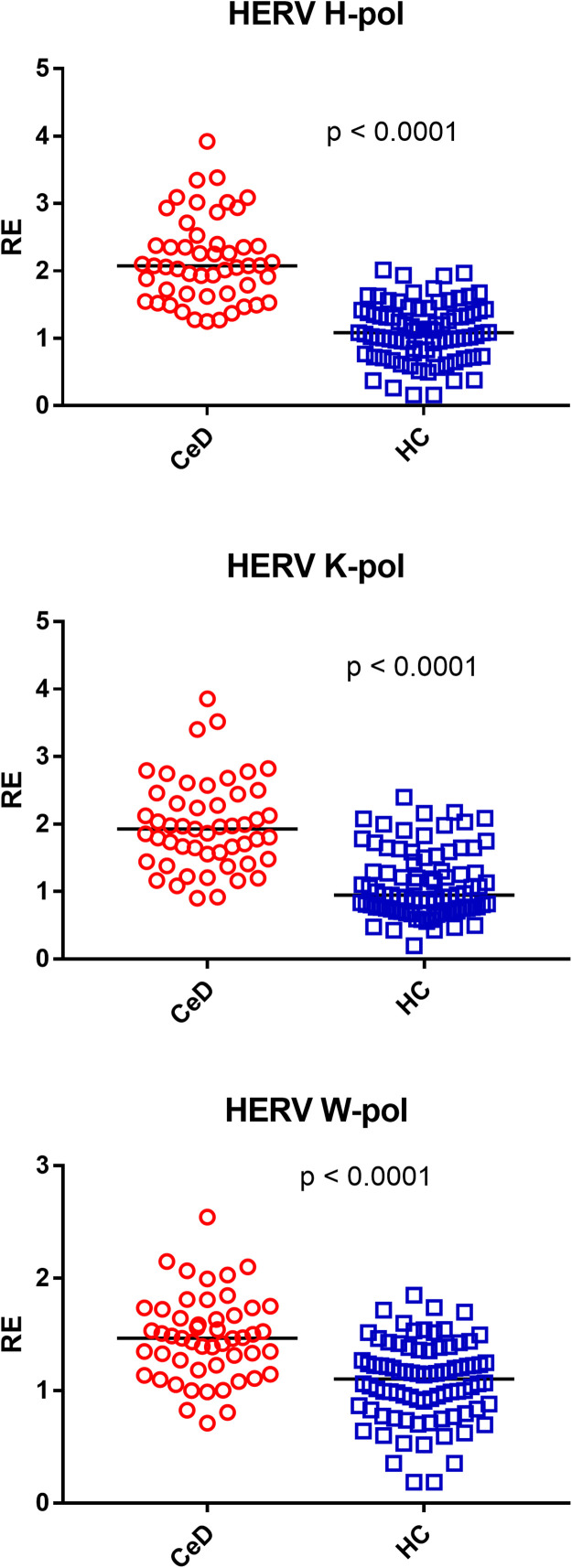
Transcription levels of the pol genes of HERV-H, HERV-K, and HERV-W in the whole blood from 51 patients with celiac disease (CeD) and 85 healthy controls (HC). RE = relative expression according to the 2−∆∆Ct method. Circles and squares show the median of three individual measurements; horizontal lines represent the median values. The p-values represent the result of the Mann–Whitney test.

As detailed in Fig. 1, the transcriptional levels of HERV-H-pol, HERV-K-pol, and HERV-W-pol were significantly higher in CeD patients than in HC. The medians and interquartile ranges (IQR 25–75 %) are reported in Table 2.

**Table 2:** Median and interquartile range (IQR 25–75 %) of transcript levels for target genes in peripheral blood from celiac disease patients (CeD) and healthy controls (HC).

Gene	Group	Median	IQR (25–75 %)
**HERV-H-pol**	CeD	2.08	1.66 – 2.39
	HC	1.08	0.78 – 1.38
**HERV-K-pol**	CeD	1.93	1.52 – 2.38
	HC	0.95	0.75 – 1.44
**HERV-W-pol**	CeD	1.47	1.21 – 1.70
	HC	1.10	0.83 – 1.36
**Syncytin 1**	CeD	1.72	0.13 – 2.47
	HC	0.99	0.71 – 1.37
**Syncytin 2**	CeD	1.43	1.08 – 1.72
	HC	0.90	0.69 – 1.32
**HERV-W-env**	CeD	1.74	1.35 – 2.10
	HC	0.93	0.74 – 1.33
**TRIM28**	CeD	1.42	1.27 – 1.61
	HC	0.99	0.78 – 1.22
**SETDB1**	CeD	1.74	1.43 – 1.93
	HC	0.93	0.78 – 1.32

### 3.3 Transcription levels of Syncytin 1, Syncytin 2, and HERV-W-env in the whole blood of patients with celiac disease (CeD) and healthy controls (HC)

**Fig. 2:**
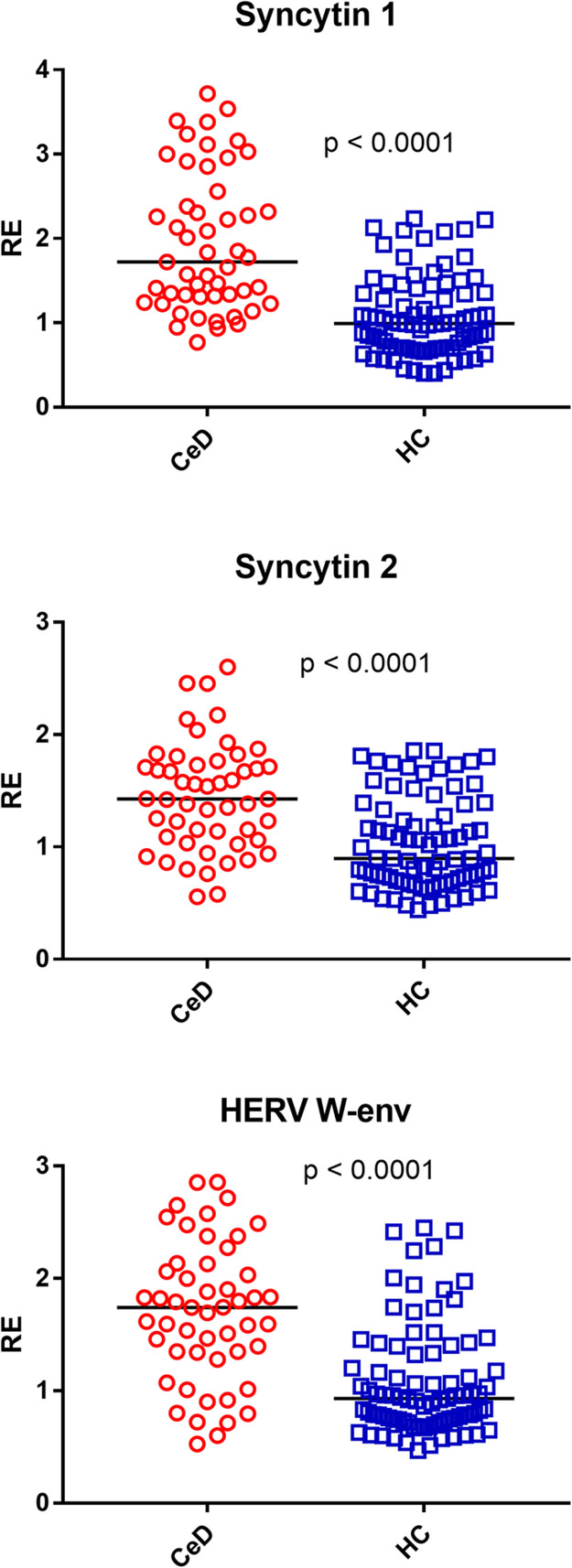
Transcription levels of Syncytin 1, Syncytin 2, and HERV-W-env in the whole blood from 51 patients with celiac disease (CeD) and 85 healthy controls (HC). RE = relative expression according to the 2−∆∆Ct method. Circles and squares show the median of three individual measurements; horizontal lines represent the median values. The p-values represent the result of the Mann–Whitney test.

The median values of the env genes of SYN1, SYN2, and HERV-W were significantly higher in CeD patiens as compared to HC (Fig. 2). The medians and IQR 25–75 % are indicated in Table 2.

### 3.4 Transcription levels of TRIM28 and SETDB1 in adults with celiac disease (CeD) and healthy controls (HC)

**Fig. 3:**
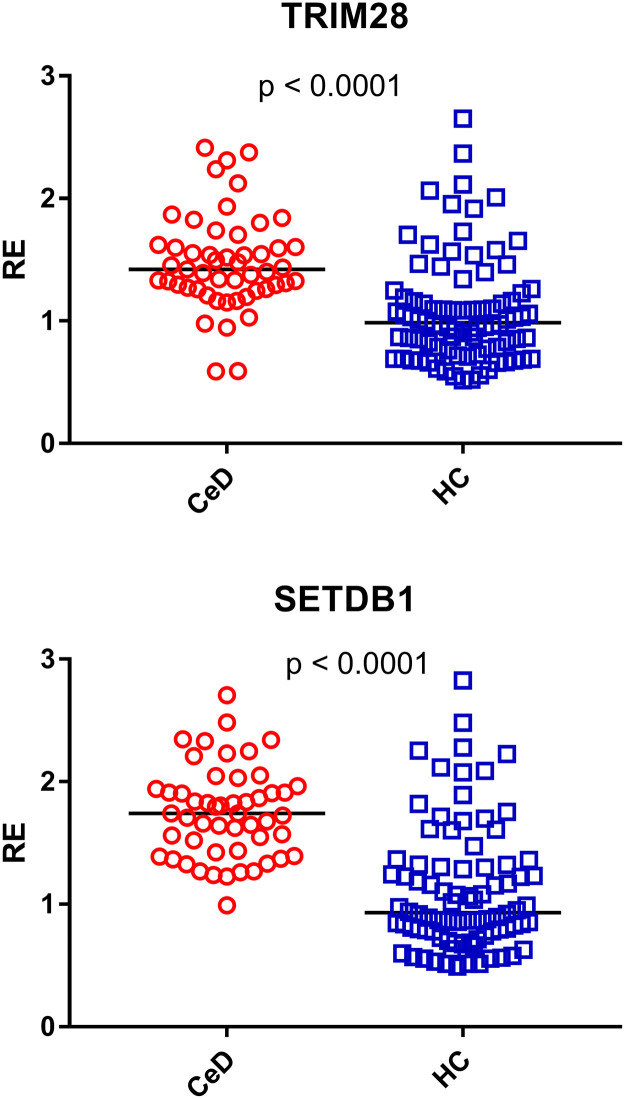
Transcription levels of TRIM28 and SETDB1 in the whole blood from 51 patients with celiac disease (CeD) and 85 healthy controls (HC). RE = relative expression according to the 2-ΔΔCt method. Circles and squares show the median of three individual measurements; horizontal lines represent the median values. The p-values represent the result of the Mann–Whitney test.

As illustrated in Fig. 3, the median transcript levels of TRIM28 and SETDB1 were significantly higher in CeD patients than in HC. Their medians and interquartile ranges (IQR 25–75 %) are reported in Table 2.

### 3.5 Correlations between expressions of TRIM28 and HERVs in celiac disease patients (CeD) and healthy controls (HC)

**Fig. 4:**
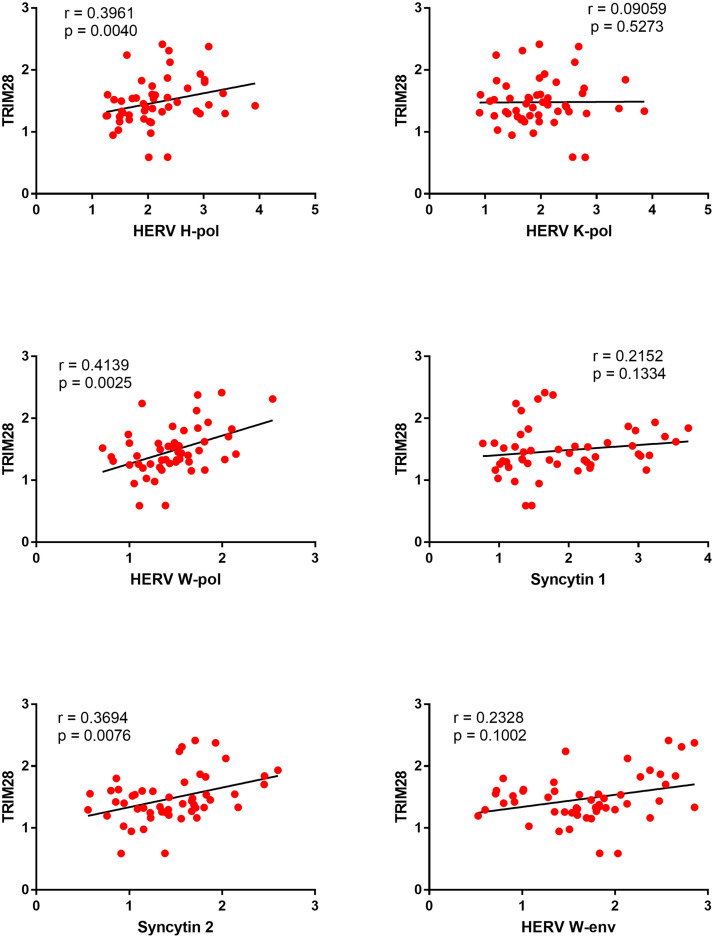
Correlations between transcription levels of TRIM28 and HERVs in whole blood from 51 celiac disease patients (CeD). RE: Relative Expression. Circles show the mean of three individual measurements. Line: Linear regression line. Statistical analysis: Spearman correlation test.

In CeD patients significant positive correlations were found between the RNA levels of TRIM28 and HERV-H-pol, HERV-W-pol, and SYN2, while no correlations emerged for HERV-K-pol, SYN1, and HERV-W-env (Fig. 4).

**Fig. 5:**
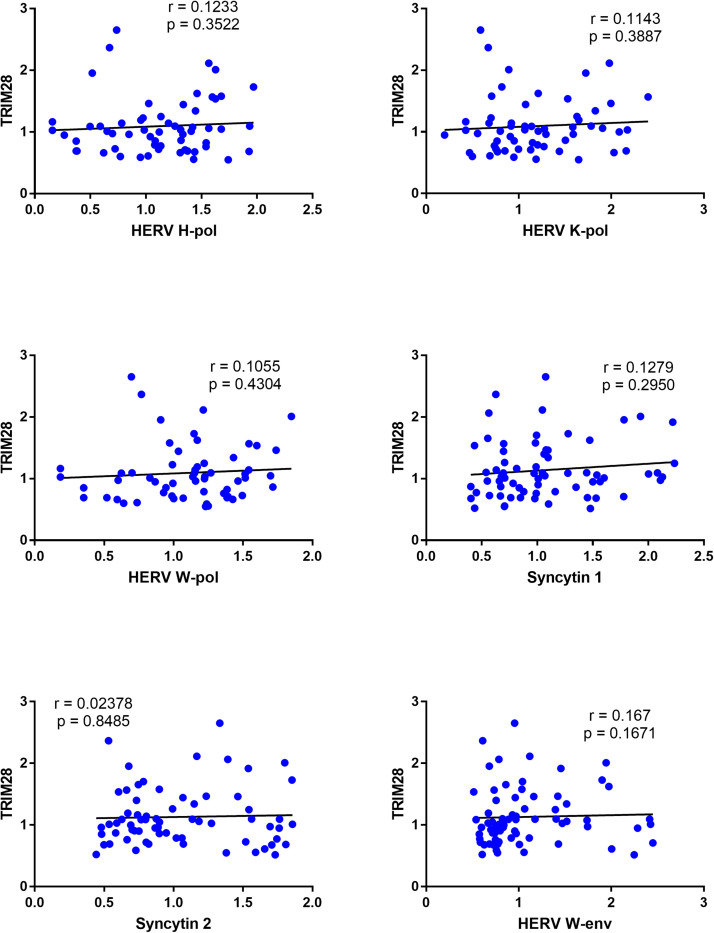
Correlations between transcription levels of TRIM28 and HERVs in healthy controls (HC). RE: Relative Expression. Circles show the mean of three individual measurements. Line: Linear regression line. Statistical analysis: Spearman correlation test.

No significant correlations were observed between the transcription levels of TRIM28 and HERVs in HC simultaneously tested for all variables (Fig. 5).

### 3.6 Correlations between expressions of SETDB1 and HERVs in CeD patients and healthy controls

**Fig. 6:**
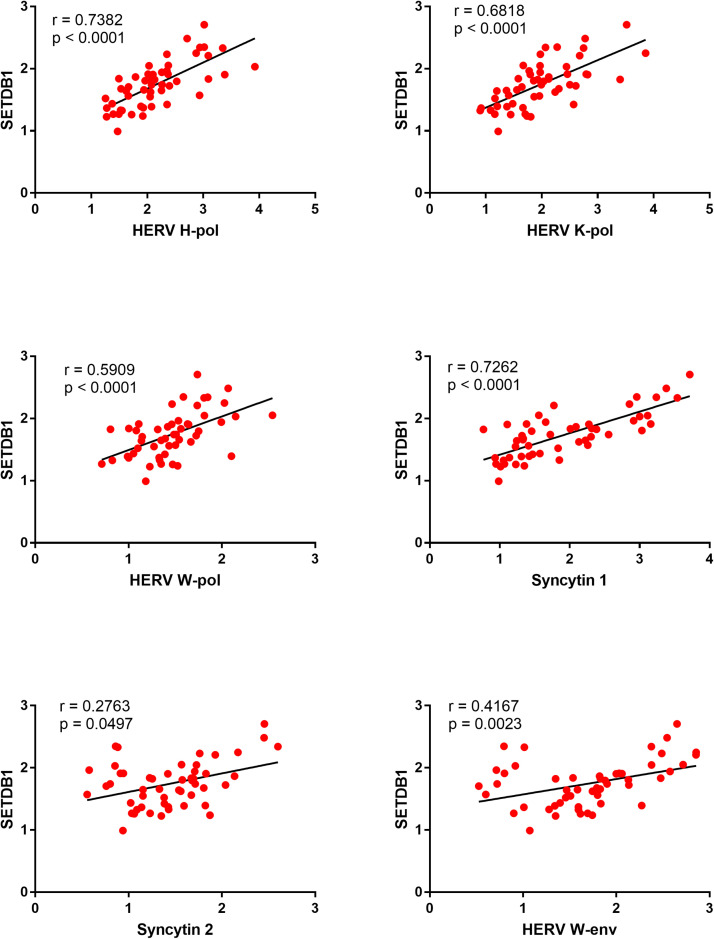
Correlations between transcription levels of SETDB1 and HERVs in whole blood from 51 CeD patients. RE: Relative Expression. Circles show the mean of three individual measurements. Line: Linear regression line. Statistical analysis: Spearman correlation test.

In CeD patients significant positive correlations were found between the expression of SETDB1 and every HERV gene, with high r values for HERV-H-pol, HERV-K-pol, HERV-W-pol, and SYN1 (Fig. 6).

**Fig. 7:**
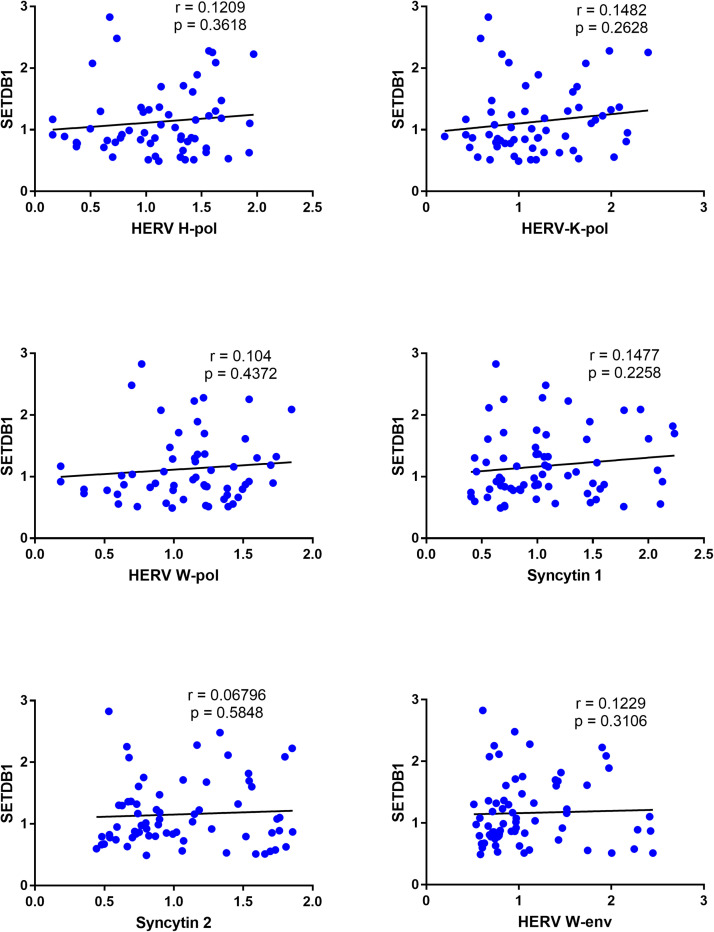
Correlations between transcription levels of SETDB1 and HERVs in healthy controls. RE: Relative Expression. Circles show the mean of three individual measurements. Line: Linear regression line. Statistical analysis: Spearman correlation test.

In contrast, no significant correlations were found in HC simultaneously tested for all variables (Fig. 7).

### 3.7 Expressions of HERVs, TRIM28, and SETDB1 in CeD patients with and without steatotic liver damage

**Fig. 8:**
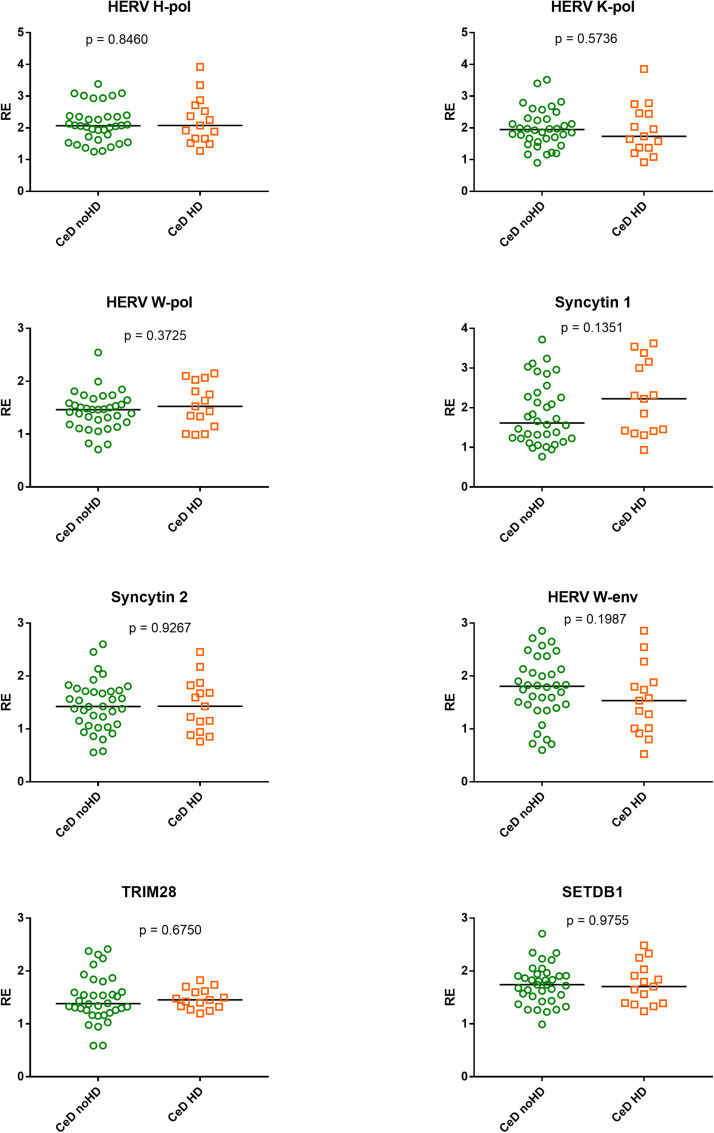
Expressions of HERVs, TRIM28 and SETDB1 in 36 CeD patients without hepatic damage (noHD) and 15 CeD patients with steatotic or fibrotic liver damage (13 steatosis and 2 fibrosis). RE = relative expression according to the 2-ΔΔCt method. Circles and squares show the median of three individual measurements; horizontal lines represent the median values. The p-values represent the result of the Mann–Whitney test.

No statistical differences were found in the RNA levels of every target gene between CeD patients with liver steatosis or fibrosis and those without liver damage (Fig. 8).

## 4 Discussion

Present results show for the first time that adults affected by CeD on GFD exhibit significantly increased transcriptional levels of HERV-H-pol, HERV-K-pol, HERV-W-pol, SYN1, SYN2, and HERV-W-env in the peripheral blood as compared with HC of similar age. This confirms our previous report of higher expression of HERV-H-pol, HERV-K-pol, and HERV-W-pol in CeD children (Tovo et al., 2021). However, whereas these were studied at diagnosis when they were constantly exposed to gluten-containing foods, current adult patients were tested after years of gluten-free diet with optimal adherence, as shown by absence of intestinal symptoms, negative anti-tTG-IgA antibody, and the results of the Biagi’s score (Biagi et al., 2009). Noteworthy, our findings are consistent with the increased antibody response against retroviral antigens recently reported in adults with CeD (Bo et al., 2024).

The underlying biological pathways leading to the persistent heightened transactivation of HERVs in CeD patients remain elusive. TRIM28 and SETDB1 are implicated in HERV silencing (Adoue et al., 2019; Turelli et al., 2014,). The HERV upregulation cannot however be ascribed to impaired transcription of TRIM28 or SETDB1, since their expressions were enhanced in our patients. Indeed, TRIM28 and SETDB1 are crucial for keeping endogenous retroviruses in a silent state in pluripotent stem cells (Fukuda and Shinkai, 2020; Turelli et al., 2014). In contrast, when these cells differentiate, the HERV activation becomes independent of such repressors (Wiznerowicz et al., 2007), which may also act as transcriptional activators (Randolph et al., 2022; Santoni de Sio et al., 2012). This possibility is supported by the positive correlations between the transcriptional levels of TRIM28/SETDB1 and HERVs, though other genes acting upstream could elicit their parallel activation.

There are mutual interactions between gut microbiota and endogenous retroviruses (Lima-Junior et al., 2021). These are not transcribed in the gut of germ-free mice, while exposure to microorganisms and their products elicit retroviral activation (Panova et al., 2020). Quantitative and qualitative changes of gut microbiota have clearly been demonstrated in CeD patients (Francavilla et al., 2023). Noteworthy, GFD fails to restore eubiosis in the digestive tract and has a negative impact on microbiome homeostasis (Wu et al., 2021). This persistent intestinal dysbiosis could contribute to the HERV overexpression observed in our patients despite the GFD, with negative effects on intestinal inflammation (Lima-Junior et al., 2021) and the risk of systemic comorbidities (Mousa et al., 2022). Targeted microbiota therapy, using precision probiotics, raises intriguing potential applications (Saviano et al., 2023), that could influence the HERV hyperexpression too.

CeD patients show enhanced release of pro-inflammatory cytokines and systemic and intestinal immune stimulation. Inflammatory cytokines lead to the proteasome-driven activation of NF-kB. After its translocation into the nucleus, it binds to specific retroviral sequences, triggering their transcription (Manghera et al., 2016). It must be underlined that HERVs can, in turn, evoke inflammatory and immune responses and fuel vigorous pathogenetic actions (Balada et al., 2010; Chuong et al., 2016; Curtin et al., 2018; Grandi and Tramontano, 2018; Rolland et al., 2006; Tovo et al., 2024). Actually, HERVs can act as promoters or enhancers of cellular genes (Chuong et al., 2016). Their RNAs can be retrotranscribed and reintegrated, through a copy-and-paste mechanism, into the DNA, causing possible mutations. The recognition of HERV RNAs as non-self by nucleic acid-sensing Toll-like receptors (TLRs) may lead to activation of the inflammasome. For instance, HERV-K stimulates the NF-kB pathway through TLR8 (Dembny et al., 2020), SYN1 activates TLR3 signal cascade (Wang et al., 2018), and HERV-W-env evokes cytokine production through TLR4 (Rolland et al., 2006). The final result may be a vicious circle leading to chronic inflammatory and altered immune reactions, that could persist regardless of gluten exposure.

CeD patients may manifest co-occurrence of a large array of disorders and many of these display HERV upregulation. GFD mitigates, but not abolish the risk of these comorbidities. Autoimmune diseases are among the most frequent conditions (Zingone et al., 2024), and they are typically associated with heightened HERV expressions (Balada et al., 2010; Curtin et al., 2018; Grandi and Tramontano, 2018; Rolland et al., 2006; Tovo et al., 2024). For instance, type 1 diabetes (T1D) is characterized by upregulation of HERVs, with HERV-W-env being detectable in pancreatic cells of 75 % of postmortem examinations (Curtin et al., 2018). CeD and T1D share the same genetic predisposition of HLA DQ2/DQ8, but, as said, the great majority of individuals with these haplotypes do no develop either disease. In contrast, affected patients share the HERV overexpression. A wealth of clinical and experimental studies supports the key role of endogenous retroviruses in development of malignancies (Stricker et al., 2023). CeD patients are at risk of intestinal neoplasms and oropharyngeal cancers and the protective effect of GFD on these CeD-related malignancies is controversial (Wang et al., 2021). Retroviral activation is increasing reported in patients with neurological and neuropsychiatric manifestations (Duarte et al., 2024). CeD subjects are at risk for neurological, neuropsychiatric and psychological problems, such as depression, foggy mind, ataxia, anxiety, and eating disorders (Clappison et al., 2020). As regards the liver steatosis, often detected in CeD and present in about 30 % of our patients, this was not associated with aberrant expressions of HERVs and/or TRIM28/SETDB1, arguing against their potential role in predisposing to fatty liver degeneration.

There is evidence that derailed epigenetic processes contribute to the development of CeD (Ghosh et al., 2022; Gnodi et al., 2022). The role of HERVs and TRIM28/SETDB1 in the regulation of epigenetic procedures is well-defined (Adoue et al., 2019; Buttler and Chuong, 2022). The high transcription levels of the latter in our patients are the first specific molecular alterations identified in CeD subjects. TRIM28 and SETDB1 affect a large number of biological functions, including the immune response, (Chikuna et al., 2021; Huang et al.; 2013) and SETDB1 participates in intestinal epithelial differentiation and in the control of local inflammation (Južnić et al., 2021; Panova et al., 2020). All these findings suggest that the aberrant expressions of TRIM28/SETDB1 in CeD patients might influence the epigenetic differentiation, expansion, and function of immunity towards a peculiar reactivity in genetically pre-disposed individuals.

The prevalence of CeD has been increasing in the last decades worldwide (Kang et al., 2013). The reason(s) remains questionable. Persistent organic pollutants, given their impact on the endocrine and immune systems, are suggested to be plausible contributors to CeD (Gaylord et al., 2020). Several lines of research show that intestinal dysbiosis precedes and accompanies the loss of gluten tolerance (Cristofori et al., 2018; Yemula, 2024). Type of diet, use of drugs, and lifestyle can affect microbiome composition and are involved in development of CeD (Panova et al., 2020). Notably, pollution (Azébi et al., 2019), cigarette smoking (Gabriel et al., 2010), nutritional changes linked to lifestyle (Pathak and Feil, 2018), and gut microbiota (Lima-Junior et al., 2021; Panova et al., 2020) can modulate retroviral expression. As mentioned, epigenetic alterations are seen as potential contributors to CeD and HERVs and TRIM28/SETDB1 may play crucial roles in epigenetic processes. Therefore, environmental factors, thought to be implicated in the recent increasing risk of CeD, could exert their actions via HERV- and/or TRIM28/SETDB1-driven changes in peculiar biologic pathways.

No significant differences were found between males and females for every variable taken into consideration both in patients and in the control group, pointing to the not relevant role of HERVs and TRIM28/SETDB1 in determining the higher risk of CeD among women, whose origin has been ascribed to genetic, hormonal, and immunological differences between the genders (Lebwohl et al., 2018).

This study has a few limitations. As for most similar studies it does not clarify whether the enhanced HERV expression is cause or secondary phenomenon of the disease. To avoid invasive procedures the analyses were performed in peripheral blood, not in intestinal biopsies. This allowed us to document persistent systemic effects of CeD. We could not however assess the expression of the same variables in the gastrointestinal mucosa nor evaluate whether there were signs of local inflammation and immune reactions.

In conclusion, what was more surprising in our study was the magnitude of upregulation and the extensive involvement of all retroviruses and cellular genes despite the long-lasting GFD. Indeed, a degree of villous atrophy was still evident in a significant proportion of CeD patients who were re-biopsied 12 months after GFD (Penny et al., 2021). Furthermore, increased levels of pro-inflammatory cytokines and immune cells persisted in the intestinal mucosa of CeD patients even after prolonged GFD; this was partly explained by the poor adherence to a correct diet (Villafuerte-Galvez et al., 2015). Although a potential exposure to gluten cannot be totally ruled out, our patients were however selected for the optimal dietary compliance, supported by lack of intestinal symptoms, negative anti-tTG-IgA, and positive answer to the Biagi’s questionnaire. The gluten-driven inflammatory condition should thus be minimal, if any, whereas the hyperstimulation of HERVs and TRIM28/SETDB1 was highly significant. This suggests that endogenous retroviruses and epigenetic regulators might be main actors in the pathophysiology of CeD. Their upregulation could account for the reduced, but persistent risk in CeD patients of developing, despite GFD, disorders typically associated with epigenetic alterations and aberrant HERV expressions, such as autoimmune diseases, neuropsychiatric manifestations, and cancers.

In this context, it must be remembered that several anti-HERV therapeutic measures might be adopted through specific trials in patients poorly responsive to GFD or with severe comorbidities, such as monoclonal antibodies, cytotoxic T lymphocytes against HERV antigens, specific anti-RNAs, and antiretroviral treatments (Garcia-Montojo et al., 2021; Hartung et al., 2022; Vermeire et al., 2023). Finally, epigenetic changes are increasingly indicated as potential therapeutic targets, using modulation of the microbiota by various nutrients (Ferenc et al., 2024) and small molecule compounds (Garcia-Martinez et al., 2021).

The following supporting information can be downloaded at: https://www……. Figure S1: Expressions of HERVs, TRIM28 and SETDB1 in males and females of healthy controls. Figure S2: Expressions of HERVs, TRIM28 and SETDB1 in males and females of patients affected by CeD.

## Funding

The research was supported by the CRT Foundation (Fondazione Cassa di Risparmio di Torino) and by the University of Turin.

## Ethics statement

The study protocol was approved by the Ethical Committee “Città della Salute e della Scienza di Torino – A.O. Ordine Mauriziano di Torino - A.S.L. Città di Torino” (code 00,696,775, 06 June 2023). All subjects enrolled in the study gave their written informed consent.

## CRediT authorship contribution statement

**Pier-Angelo Tovo:** Writing – original draft, Conceptualization. **Angelo Armandi:** Writing – review & editing, Writing – original draft, Data curation. **Mauro Bruno:** Resources. **Gian Paolo Caviglia:** Data curation. **Paola Montanari:** Formal analysis. **Demis Pitoni:** Resources. **Cristina Calvi:** Formal analysis, Data curation. **Simone Frara:** Resources. **Eleonora Dileo:** Resources. **Stefano Gambarino:** Data curation. **Ilaria Galliano:** Writing – review & editing, Data curation. **Davide Giuseppe Ribaldone:** Writing – review & editing, Writing – original draft, Conceptualization. **Massimiliano Bergallo:** Writing – review & editing, Writing – original draft, Supervision, Conceptualization.

## Declaration of competing interest

The authors declare no conflicts of interest.

## Appendix Supplementary materials

Image, application 1

## References

[bib0001] Adoue V., Binet B., Malbec A. (2019). The histone methyltransferase SETDB1 controls T helper cell lineage integrity by repressing endogenous retroviruses. Immunity.

[bib0002] Azébi S., Batsché E., Michel F. (2019). Expression of endogenous retroviruses reflects increased usage of atypical enhancers in T cells. EMBO J..

[bib0003] Balada E., Vilardell-Tarrés M., Ordi-Ros J. (2010). Implication of human endogenous retroviruses in the development of autoimmune diseases. Intern. Rev. Immunol..

[bib0004] Biagi F., Andrealli A., Bianchi P.I. (2009). A gluten-free diet score to evaluate dietary compliance in patients with coeliac disease. Br. J. Nutr..

[bib0005] Bo M., Manetti R., Biggio M.L. (2024). The humoral immune response against human endogenous retroviruses in celiac disease: a case-control study. Biomedicines.

[bib0006] Buttler C.A., Chuong E.B. (2022). Emerging roles for endogenous retroviruses in immune epigenetic regulation. Immunol.

[bib0007] Cazac G.D., Mihai B.M., Ștefănescu G. (2024). Celiac disease, gluten-free diet and metabolic dysfunction-associated steatotic liver disease. Nutrients.

[bib0008] Chikuma S., Yamanaka S., Nakagawa S. (2021). TRIM28 expression on dendritic cells prevents excessive T cell priming by silencing endogenous retrovirus. J. Immunol..

[bib0009] Chuong E.B., Elde N.C., Feschotte C. (2016). Regulatory evolution of innate immunity through co-option of endogenous retroviruses. Science.

[bib0010] Clappison E., Hadjivassiliou M., Zis P. (2020). Psychiatric manifestations of coeliac disease, a systematic review and meta-analysis. Nutrients.

[bib0011] Cristofori F., Indrio F., Miniello V.L. (2018). Probiotics in celiac disease. Nutrients.

[bib0012] Curtin F., Bernard C., Levet S. (2018). A new therapeutic approach for type 1 diabetes: rationale for GNbAC1, an anti-HERV-W-Env monoclonal antibody. Diabetes. Obes. Metab..

[bib0013] Dembny P., Newman A.G., Singh M. (2020). Human endogenous retrovirus HERV-K(HML-2) RNA causes neurodegeneration through toll-like receptors. JCI. Insight..

[bib0014] Duarte R.R.R., Pain O., Bendall M.L. (2024). Integrating human endogenous retroviruses into transcriptome-wide association studies highlights novel risk factors for major psychiatric conditions. Nat. Commun..

[bib0015] Fasano A., Catasi C. (2012). Clinical practice. Celiac disease. N. Engl. J. Med..

[bib0016] Ferenc K., Sokal-Dembowska A., Helma K. (2024). Modulation of the gut microbiota by nutrition and its relationship to epigenetics. Int. J. Mol. Sci..

[bib0017] Francavilla A., Ferrero G., Pardini B. (2023). Gluten-free diet affects fecal small non-coding RNA profiles and microbiome composition in celiac disease supporting a host-gut microbiota crosstalk. Gut. Microbes..

[bib0018] Fukuda F.K., Shinkai Y. (2020). SETDB1-mediated silencing of retroelements. Viruses.

[bib0019] Gabriel U., Steidler A., Trojan L. (2010). Smoking increases transcription of human endogenous retroviruses in a newly established in vitro cell model and in normal urothelium. AIDS Res. Hum. Retrovir..

[bib0020] Garcia-Martinez L., Zhang Y., Nakata Y. (2021). Epigenetic mechanisms in breast cancer therapy and resistance. Nat. Commun..

[bib0021] Garcia-Montojo M., Fathi S., Norato G. (2021). Inhibition of HERV-K (HML-2) in amyotrophic lateral sclerosis patients on antiretroviral therapy. J. Neurol. Sci..

[bib0022] Garcia-Montojo M., Rodriguez-Martin E., Ramos-Mozo P. (2020). Syncytin-1/HERV-W envelope is an early activation marker of leukocytes and is upregulated in multiple sclerosis patients. Eur. J. Immunol..

[bib0023] Gaylord A., Trasande L., Kannan K. (2020). Persistent organic pollutant exposure and celiac disease: a pilot study. Env. Res..

[bib0024] Ghosh S., Khetarpal P., Senapati S. (2022). Functional implications of the CpG island methylation in the pathogenesis of celiac disease. Mol. Biol. *Re.*.

[bib0025] Gnodi E., Meneveri R., Barisani D. (2022). Celiac disease: from genetics to epigenetics. World J. Gastroenterol..

[bib0026] Grandi N., Tramontano E. (2018). Human endogenous retroviruses are ancient acquired elements still shaping innate immune responses. Front Immunol..

[bib0027] Huang C., Martin S., Pfleger C. (2013). Cutting Edge: a novel, human-specific interacting protein couples FOXP3 to a chromatin-remodeling complex that contains KAP1/TRIM28. J. Immunol..

[bib0028] Hartung H.-P., Derfuss T., Cree B.A. (2022). Efficacy and safety of Temelimab in multiple sclerosis: results of a randomized phase 2b and extension study. Mult. Scler..

[bib0029] Johnson W.E. (2019). Origins and evolutionary consequences of ancient endogenous retroviruses. Nat. Rev. Microbiol..

[bib0030] Južnić L., Peuker K., Strigli A. (2021). SETDB1 is required for intestinal epithelial differentiation and the prevention of intestinal inflammation. Gut.

[bib0031] Kang J.Y., Kang A.H., Green A. (2013). Systematic review: worldwide variation in the frequency of coeliac disease and changes over time. Aliment. Pharmacol. Ther..

[bib0032] Lebwohl B., Sanders D.S., Green P.H.R. (2018). Celiac disease. Lancet.

[bib0033] Lima-Junior D.S., Krishnamurthy S.R., Bouladoux N. (2021). Endogenous retroviruses promote homeostatic and inflammatory responses to the microbiota. Cell.

[bib0034] Livak K.J., Schmittgenb T.D. (2001). Analysis of relative gene expression data using real-time quantitative PCR and the 2-DDCT method. Method.

[bib0035] Lokossou A.G., Toudic C., Nguyen P.T. (2020). Endogenous retrovirus-encoded Syncytin-2 contributes to exosome-mediated immunosuppression of T cells. Biol. Reprod..

[bib0036] Manghera M., Douville R.N. (2013). Endogenous retrovirus-K promoter: a landing strip for inflammatory transcription factors?. Retrovirology.

[bib0037] Manghera M., Ferguson-Parry J., Lin R. (2016). NF-κb and IRF1 induce endogenous retrovirus K expression via interferon-stimulated response elements in its 5’ long terminal repeat. J. Virol..

[bib0038] Mousa W.K., Chehadeh F., Husband S. (2022). Microbial dysbiosis in the gut drives systemic autoimmune diseases. Front. Immunol..

[bib0039] Panova V., Attig J., Young G.R. (2020). Antibody-induced internalisation of retroviral envelope glycoproteins is a signal initiation event. PLoS. Pathog..

[bib0040] Pathak R., Feil R. (2018). Environmental effects on chromatin repression at imprinted genes and endogenous retroviruses. Curr. Opin. Chem. Bio..

[bib0041] Penny H.A., Raju S.A., Lau M.S. (2021). Accuracy of a no-biopsy approach for the diagnosis of coeliac disease across different adult cohorts. Gut.

[bib0042] Randolph K., Hyder U., D’Orso I. (2022). KAP1/TRIM28: transcriptional activator and/or repressor of viral and cellular programs?. Front. Cell Infect. Microbiol..

[bib0043] Rolland A., Jouvin-Marche E., Viret C. (2006). The envelope protein of a human endogenous retrovirus-W family activates innate immunity through CD14/TLR4 and promotes Th1-like responses. J. Immunol..

[bib0044] Rubio-Tapia A., Hill I.D., Semrad C. (2023). American college of gastroenterology guidelines update: diagnosis and management of celiac disease. Am. J. Gastroenterol..

[bib0045] Santoni de Sio F.R., Barde I., Offner S. (2012). KAP1 regulates gene networks controlling t-cell development and responsiveness. FASEB J..

[bib0046] Saviano A., Petruzziello C., Brigida M. (2023). Gut microbiota alteration and its modulation with probiotics in celiac disease. Biomedicines.

[bib0047] Stricker E., Peckham-Gregory E.C., Scheurer M.E. (2023). HERVs and cancer- a comprehensive review of the relationship of human endogenous retroviruses and human cancers. Biomedicines.

[bib0048] Tovo P.A., Monti G., Daprà V. (2022). Enhanced expression of endogenous retroviruses and of TRIM28 and SETDB1 in children with food allergy. Clin. Transl. Allergy.

[bib0049] Tovo P.A., Opramolla A., Pizzol A. (2021). Overexpression of endogenous retroviruses in children with celiac disease. Eur. J. Pediat..

[bib0050] Tovo P.A., Ribaldone D.G., Galliano I. (2024). Enhanced transcription of human endogenous retroviruses and TRIM28 downregulation in patients with inflammatory bowel disease. Viruses..

[bib0051] Tovo P.A., Ribaldone D.G., Caviglia G.P. (2025). Patients with irritable bowel syndrome exhibit aberrant expression of endogenous retroviruses and SETDB1. Cells.

[bib0052] Turelli P., Castro-Diaz N., Marzetta F. (2014). Interplay of TRIM28 and DNA methylation in controlling human endogenous retroelements. Genome Res..

[bib0053] Vermeire S., Solitano V., Peyrin-Biroulet L. (2023). Obefazimod: a first-in-class drug for the treatment of ulcerative colitis. J. Crohns. Colitis..

[bib0054] Villafuerte-Galvez J., Vanga R.R., Dennis M. (2015). Factors governing long-term adherence to a gluten-free diet in adult patients with coeliac disease. Aliment. Pharmacol. Ther..

[bib0055] Wang C., Xia Z., Li Z. (2022). Expression of SET domain bifurcated histone lysine methyltransferase 1 and its clinical prognostic significance in hepatocellular carcinoma. J. Clin. Lab. Ana..

[bib0056] Wang M., Yu M., Kong W.J. (2021). Association between intestinal neoplasms and celiac disease: a review. World J. Gastrointest. Oncol..

[bib0057] Wang X., Liu Z., Wang P. (2018). Syncytin-1, an endogenous retroviral protein, triggers the activation of CRP via TLR3 signal cascade in glial cells. Brain Behav. Immun..

[bib0058] Weber M., Padmanabhan Nair V., Bauer T. (2021). Increased HERV-K(HML-2) transcript levels correlate with clinical parameters of liver damage in Hepatitis C patients. Cells.

[bib0059] Wiznerowicz M., Jakobsson J., Szulc J. (2007). The Kruppel-associated box repressor domain can trigger de novo promoter methylation during mouse early embryogenesis. J. Biol. Chem..

[bib0060] Wu X., Qian L., Liu K. (2021). Gastrointestinal microbiome and gluten in celiac disease. Ann. Med..

[bib0061] Yemula N. (2024). Gut microbiota in celiac disease. Ann. Gastroenterol..

[bib0062] Zingone F., Bai J.C., Cellier C. (2024). Celiac disease-related conditions: who to test?. Gastroenterology.

