# Supplementary material for: Adults with celiac disease exhibit overexpression of endogenous retroviruses, TRIM28, and SETDB1 despite gluten-free diet
Source: Virus Res. 2025 Aug 5;359:199613. doi: 10.1016/j.virusres.2025.199613 (PMC12357293; doi:10.1016/j.virusres.2025.199613)
Supplement: Supplementary file 1 [file mmc1.docx]

**Supplementary Information**

**Figure S1:** Expressions of HERVs, TRIM28 and SETDB1 in males and females of 85 healthy controls.


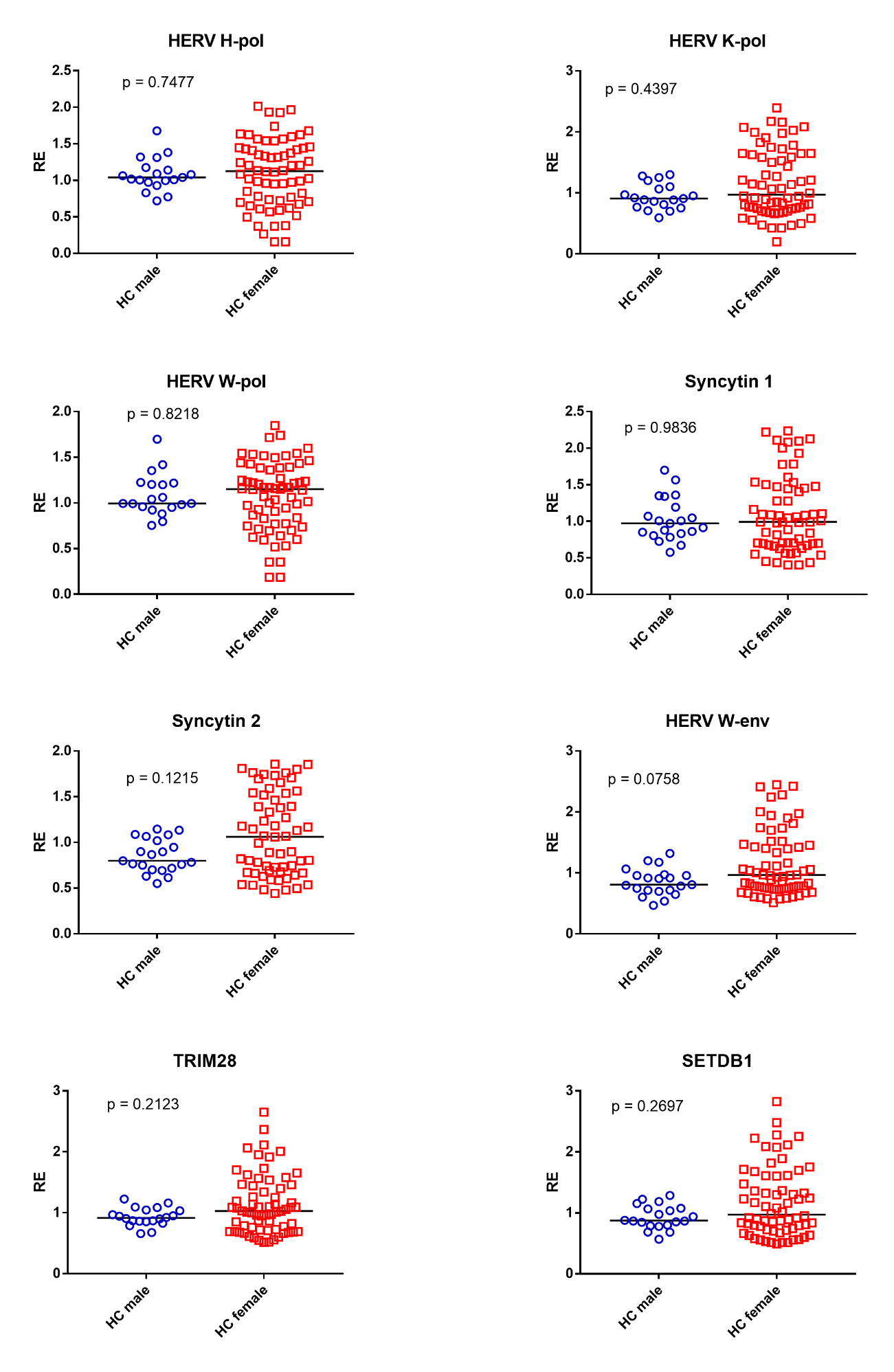


RE = relative expression according to the 2-ΔΔCt method. Circles and squares show the median of three individual measurements; horizontal lines represent the median values. The p-values represent the result of the Mann–Whitney test.

**Figure S2:** Expressions of HERVs, TRIM28 and SETDB1 in males and females of patients affected by CeD

**
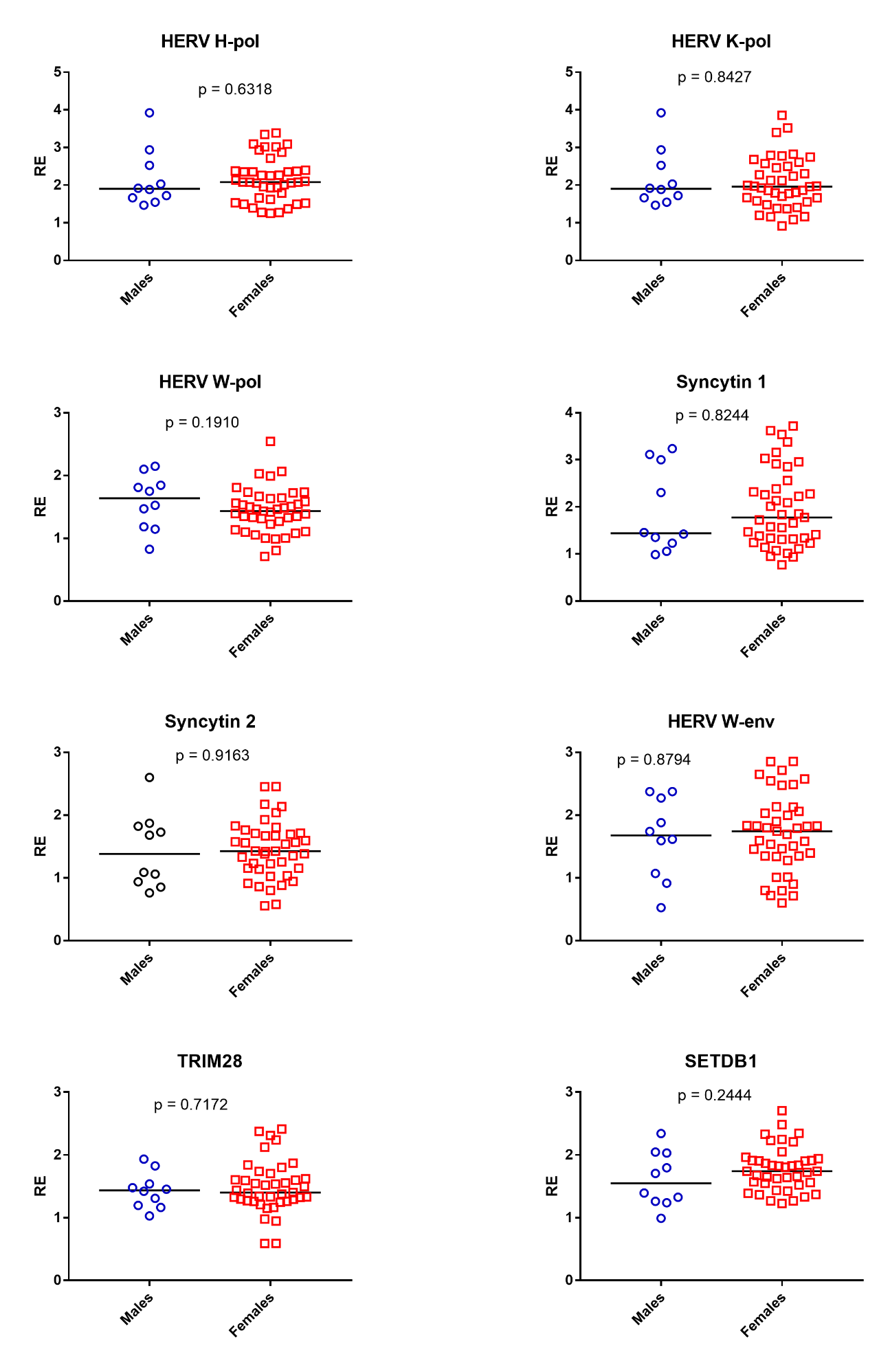
**

RE = relative expression according to the 2-ΔΔCt method. Circles and squares show the median of three individual measurements; horizontal lines represent the median values. The p-values represent the result of the Mann–Whitney test.
